# Development and validation of a nomogram (APGRC) to predict the presence of germline DNA damage repair pathogenic variants in Asian patients with prostate cancer

**DOI:** 10.1002/ctm2.1411

**Published:** 2023-09-12

**Authors:** Tingwei Zhang, Yu Wei, Boon Hao Hong, Takayuki Sumiyoshi, Enya Hui Wen Ong, Hao Zeng, Yonghong Li, Chi‐Fai Ng, Jian Pan, Bangwei Fang, Beihe Wang, Junlong Wu, Hongkai Wang, Shusuke Akamatsu, Melvin Lee Kiang Chua, Dingwei Ye, Yao Zhu

**Affiliations:** ^1^ Department of Urology Fudan University Shanghai Cancer Center Shanghai China; ^2^ Department of Oncology Shanghai Medical College Fudan University Shanghai China; ^3^ Shanghai Genitourinary Cancer Institute Shanghai China; ^4^ Division of Medical Sciences National Cancer Centre Singapore Singapore; ^5^ Department of Urology Kyoto University Graduate School of Medicine Kyoto Japan; ^6^ Department of Urology Institute of Urology West China Hospital Sichuan University Chengdu China; ^7^ Department of Urology Sun Yat‐Sen University Cancer Center State Key Laboratory of Oncology in South China, Collaborative Innovation Center for Cancer Medicine Guangzhou China; ^8^ Department of Surgery and SH Ho Urology Center Chinese University of Hong Kong Hong Kong Hong Kong; ^9^ Department of Urology Nagoya University Graduate School of Medicine Aichi Japan; ^10^ Department of Head and Neck and Thoracic Cancers Division of Radiation Oncology National Cancer Centre Singapore Singapore; ^11^ Oncology Academic Programme Duke‐NUS Medical School Singapore

Dear Editor,

Prostate cancer (PCa) is a prominent global cancer among men, estimated at 1.3 million diagnoses and 359 thousand deaths in 2018,^1^ with a strong hereditary component. DNA damage repair (DDR) genes play a vital role in maintaining genetic stability and germline mutations in them can result in high‐risk biological behaviours^2^ and influence the response to targeted therapies like platinum treatments, poly‐ADP ribose polymerase (PARP) inhibitors, and pembrolizumab.^3^ Identifying germline DDR pathogenic variants (PVs) carriers in PCa patients has significant implications for personalised treatment, risk reduction and familial testing. However, current genetic testing guidelines are primarily based on Western populations, with a lack of representation from Asians. Thus, we aimed to develop the APGRC (Asian PCa Germline Risk Calculator), a risk assessment nomogram for predicting the probability of carrying germline PVs in 14 PCa predisposition DDR genes.

The development cohort included 2,052 unselected PCa patients from four Chinese cancer centers. Stepwise logistic regression determined predictors for the development of the APGRC nomogram, validated in an independent cohort of 743 patients (347 Singaporean patients and 396 Japanese patients^4^) (Figure S1). Relevant details of model development and validation were provided in Supporting information.

The characteristics of the study participants were summarised in Table S1. The Japan cohort (*N* = 549) exhibited the most aggressive characteristics, with 406 (74.5%) participants having metastatic PCa. The Singapore cohort (*N* = 920) demonstrated predominantly early features, with 583 (63.5%) participants having localized low‐ or intermediate‐risk PCa, while the development cohort exhibited an intermediate disease profile (Table S1).

In total, 162 (7.9%), 27 (2.9%) and 35 (6.4%) participants in the development cohort, Singapore cohort and Japan cohort, respectively, carried germline PVs in the 14 PCa predisposition DDR genes (Table 1, Figure S2). Germline pathogenic/likely PVs were detailed in Table S2.

**TABLE 1:** Univariate logistic analysis of clinical characteristics between patients with and without germline DNA damage repair pathogenic variants.

Characteristics	DDR (*n* = 162)	Non‐DDR (*n* = 1890)	OR (95% CI)	*P* value
**Age at diagnosis (Median, IQR, years old)**	63.5 (58.0–68.0)	67.0 (61.0–72.0)	.96 (.94–.98)	**<.001**
**Personal history of other cancers**				
Yes	16	87	2.27 (1.26–3.87)	**.004**
No	146	1803		
**Personal history of LS‐related cancers**				
Yes	11	38	3.55 (1.70–6.86)	**<.001**
No	151	1852		
**PSA at diagnosis (Median, IQR, ng/mL)** ^*^	100.0 (26.5–225.2)	61.8 (17.3–156.7)	1.40 (1.10–1.77)	**.006**
**Gleason score**				
> 8	85	833	1.40 (1.02–1.93)	**.040**
≤8	77	1057		
**Metastasis disease at test**				
Yes	114	1088	1.75 (1.24–2.50)	**0.002**
No	48	802		
**De novo metastasis**				
Yes	96	923	1.52 (1.10–2.12)	**.011**
No	66	967		
**Stage**				
Regional/metastatic	128	1229	2.02 (1.39–3.03)	**<.001**
Localised	34	661		
**Family history of any cancer (first/second/third degree relatives)**				
Yes	47	211	3.25 (2.23–4.67)	**<.001**
No	115	1679		
**Family history of any cancer (first degree relatives)**				
Yes	45	176	3.63 (2.47–5.27)	**<.001**
No	118	1714		
**Family history of prostate cancer (first/second/third degree relatives)**				
Yes	3	32	1.10 (0.26–3.10)	**.881**
No	159	1858		
**Family history of prostate cancer (first degree relatives)**				
Yes	3	25	1.41 (0.33–4.07)	**.579**
No	159	1865		
**Family history of breast/pancreatic/ovarian cancers (first/second/third degree relatives)**				
Yes	20	19	13.87 (7.22–26.76)	**<.001**
No	142	1871		
**Family history of breast/pancreatic/ovarian cancers (first degree relatives)**				
Yes	19	13	19.18 (9.36–40.52)	**<.001**
No	143	1877		
**Family history of LS‐related cancers (first/second/third degree relatives)**				
Yes	28	75	5.06 (3.12–7.99)	**<.001**
No	134	1815		
**Family history of LS‐related cancers (first degree relatives)**				
Yes	23	61	4.96 (2.93–8.15)	**<.001**
No	139	1829		

*Notes*: Variables with *P* < .05 are presented in bold. Lynch syndrome‐related cancers: colorectal, endometrial, gastric, ovarian, small bowel, pancreatic, liver, urinary tract (renal pelvis, ureter, bladder), bile duct, brain and skin (sebaceous carcinoma, keratoacanthomas, sebaceous adenomas) cancers. De novo metastasis: metastasised at the time of initial diagnosis.

Abbreviations: CI, confidence interval; DDR, DNA damage repair; LS, Lynch syndrome; OR, odds ratio; PSA, prostate specific antigen.

* : logPSA was used in the univariate logistic analysis because of some extremums.

The results of univariate logistic analysis indicated a number of predictors of germline DDR PVs. The carriers were found to be more likely to have a younger age at diagnosis (63.5 vs. 67.0 years old, *P* < .001), higher prostate specific antigen (PSA) levels (100.0 vs. 61.8 ng/mL, *P* = 0.006), and higher Gleason scores (52.5% vs. 44.1% in > 8 group, *P* = .040) compared to non‐carriers (Table 1). Additionally, carriers were more likely to have a personal or family history of cancer, excluding PCa (Table 1). The carriers also displayed more aggressive disease characteristics, such as a higher rate of metastatic disease (70.1% vs. 57.6%, *P* = .002) and later stage of disease (Table 1).

Based on the results of the univariate logistic analysis, stepwise logistic regression showed that the most significant predictors of the presence of germline DDR PVs were age at diagnosis (OR .95 [95% CI .93 to .98]; *P* < .001), personal history of Lynch syndrome‐related cancers (OR 4.63 [95% CI 2.03–9.70]; *P* < .001), PSA at diagnosis (log, OR 1.34 [95% CI 1.02–1.76]; *P* = .037), stage (Regional/Metastatic vs. Localised, OR 2.11 [95% CI 1.35–3.39]; *P* = .001), family history of breast, pancreatic, or ovarian cancers in first‐degree relatives (OR 18.66 [95% CI 8.24–34.12]; *P* < .001), and family history of Lynch syndrome‐related cancers in first‐, second‐, or third‐ degree relatives (OR 4.12 [95% CI 2.39–6.89]; *P* < .001) (Table 2).

**TABLE 2:** Most informative variables for prediction of positive result after stepwise logistic regression.

Stepwise coefficients^*^	Estimate	OR (95% CI)	*P* value
Age at diagnosis (numeric)	−.0463	.95 (.93–.98)	<.001
Personal history of LS‐related cancers (Yes vs. No)	1.5330	4.63 (2.03–9.70)	<.001
PSA at diagnosis (log, numeric)	.2902	1.34 (1.02–1.76)	.037
Stage (Regional/Metastatic vs. Localised)	0.7485	2.11 (1.35–3.39)	.001
Family history of breast/pancreatic/ovarian cancers (first degree relatives) (Yes vs. No)	2.9261	18.66 (8.24–43.12)	<.001
Family history of LS‐related cancers (first/second/third degree relatives) (Yes vs. No)	1.4159	4.12 (2.39 –6.89)	<.001

*Notes*: Lynch syndrome‐related cancers: colorectal, endometrial, gastric, ovarian, small bowel, pancreatic, liver, urinary tract (renal pelvis, ureter, bladder), bile duct, brain and skin (sebaceous carcinoma, keratoacanthomas, sebaceous adenomas) cancers.

Abbreviations: CI, confidence interval; LS, Lynch syndrome; OR, odds ratio; PSA, prostate specific antigen.

* : The variables presented here are adjusted for all other variables in the table.

A nomogram prediction model, named APGRC, was developed based on the results of the multivariable stepwise logistic regression (Figure 1). The performance of APGRC was compared to current guidelines^5^, ^6^, ^7^, ^8^, ^9^, ^10^ (Table S3) in the development cohort, and it was found to have the highest area under the curve (AUC) value (.706 [95% CI .664–.748]), while other guidelines had lower AUC values ranging from .518 to .590 (Figure 2A, Table S4).

**FIGURE 1:**
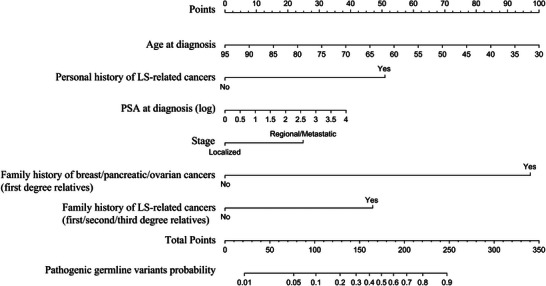
Nomogram to predict the presence of germline DDR pathogenic variants based on age at diagnosis, PSA, stage, personal and family history of cancers. *Notes*: Lynch syndrome‐related cancers: colorectal, endometrial, gastric, ovarian, small bowel, pancreatic, liver, urinary tract (renal pelvis, ureter, bladder), bile duct, brain and skin (sebaceous carcinoma, keratoacanthomas, sebaceous adenomas) cancers. Abbreviations: LS, Lynch syndrome; PSA, prostate specific antigen.

**FIGURE 2:**
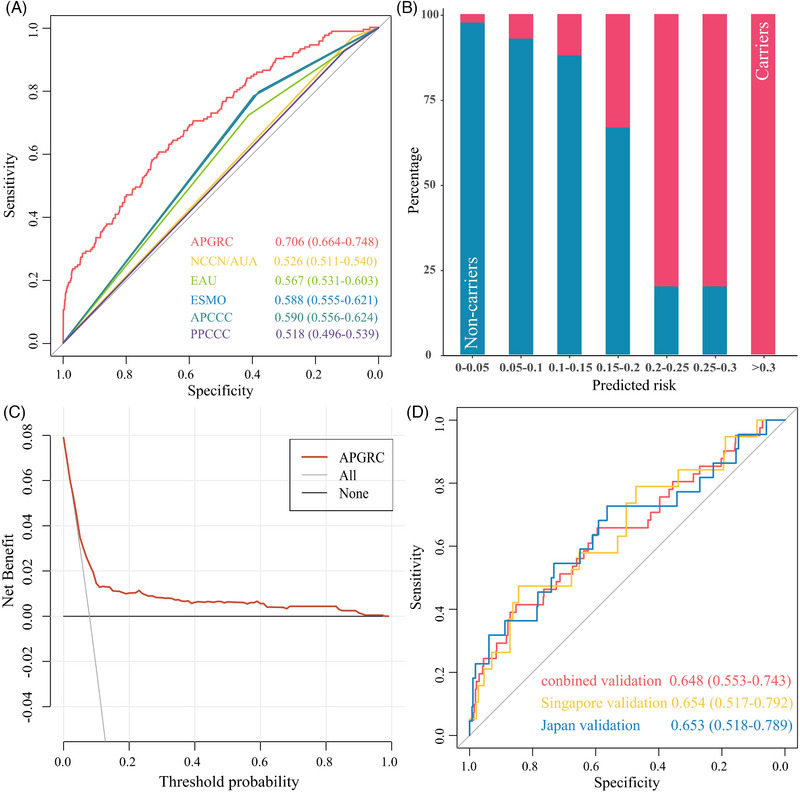
Evaluation and validation of the predictive performance of APGRC. (A) ROC curves of APGRC and current guidelines in development cohort. (B) The predicted risk generated by APGRC showed high agreement with the actual probabilities. (C) DCA demonstrating the net benefit of predicting the presence of germline pathogenic variants in development cohort. (D) ROC curves of APGRC in validation cohorts. Abbreviations: NCCN, National Comprehensive Cancer Network; AUA, American Urological Association; EAU, European Association of Urology; ESMO, European Society of Medical Oncology; APCCC, Advanced Prostate Cancer Consensus Conference; PPCCC, Philadelphia Prostate Cancer Consensus Conference; ROC, receiver operating characteristic; DCA, decision curve analysis.

The APGRC prediction model exhibited high concordance with actual probabilities, as demonstrated by the results of the predicted risk analysis (Figure 2B). Furthermore, the well‐calibrated nature of the model was verified through the Hosmer–Lemeshow test (*P* = .706). The decision curve analysis (DCA) of the development cohort revealed that using APGRC to identify patients for germline testing above a 5% probability threshold resulted in a favourable clinical impact (Figure 2C). The reduction rate in germline genetic testing and the corresponding missing rate of germline DDR PV carriers across varying threshold probabilities determined by APGRC were summarised in Table S5. Notably, at a 4.0% threshold, APGRC achieved a reduction of 11.3% (231/2 152) in germline genetic testing, missing only 1.2% (2/162) of carriers in the development cohort. Similarly, at a 3.0% threshold, APGRC exhibited a reduction of 14.1% (105/743) in germline genetic testing, albeit with a slightly higher missing rate of 4.9% (2/41) among germline DDR PV carriers in the validation cohort. Furthermore, APGRC has demonstrated commendable validation outcomes within both the early‐stage disease population, as exemplified by the Singapore cohort (.654 [95% CI.517–.792]), and the late‐stage disease population, as represented by the Japanese cohort (.653 [95% CI.518–.789]) (Figure 2D). The similar AUC values (.706 [95% CI .664–.748] vs. .648 [95% CI .553–.743]) in the combined validation cohort and the development cohort (Figure 2D) underscore APGRC's consistent discriminatory capacity.

It is worth mentioning that none of the existing guidelines take age at diagnosis into account as a criterion (Table S3). To evaluate the impact of age at diagnosis on the performance of APGRC, we excluded this factor from the model. The results showed a decline in APGRC's performance, with an AUC of .666 in predicting the presence of germline DDR PVs in the developing cohort (Figure S3B, highlighting age at diagnosis as a crucial predictive factor for the presence of germline DDR PVs.

In conclusion, the APGRC model represents a valuable tool in the prediction of the presence of germline DDR PVs in Asian patients with PCa. This is the first study to integrate various important clinical variables in a comprehensive and tailored manner for the Asian population. By doing so, APGRC can support clinical decision‐making by physicians and patients and pave the way to improve precision oncology in an underrepresented patient population.

## CONFLICTS OF INTEREST STATEMENT

Melvin L.K. Chua reports personal fees from Astellas, Janssen, Bayer, Pfizer, MSD, Varian, IQVIA, Telix Pharmaceuticals, AstraZeneca, personal fees and non‐financial support from BeiGene, non‐financial support from Decipher Biosciences, non‐financial support from MedLever, consults for immunoSCAPE Inc., and is a co‐inventor of the patent of a High Sensitivity Lateral Flow Immunoassay For Detection of Analyte in Sample (10202107837T), Singapore and serves on the Board of Directors of Digital Life Line Pte Ltd that owns the licensing agreement of the patent, outside the submitted work. The other authors declare that they have no competing interests.

## FUNDING INFORMATION

the National Natural Science Foundation of China (82172621, YZ); Chinese Anti‐Cancer Association‐Hengrui PARP Inhibitor Tumor Research Fund (Phase I, YZ); Bethune Urology Tumor Special Research Fund (mnzl202004, YZ); Program of Shanghai Academic Research Leader (23XD1420600, YZ); Shanghai Medical Innovation Research Special Project (21Y11904300, YZ); the General Program of Beijing Xisike Clinical Oncology Research Foundation (Y‐2019AZMS‐0012, YZ); the National Medical Research Council Singapore Clinician Scientist Award (NMRC/CSA‐INV/0027/2018, CSAINV20nov‐0021, MC); the Duke‐NUS Oncology Academic Program Goh Foundation Proton Research Programme (MC); NCCS Cancer Fund (MC); the Kua Hong Pak Head and Neck Cancer Research Programme (MC)

## Supporting information

Supporting Information

Supporting Information

Supporting Information

## Data Availability

All data generated or analysed during this study are included in this published article and its supplementary files.
