# Supplementary material for: Development and validation of a nomogram (APGRC) to predict the presence of germline DNA damage repair pathogenic variants in Asian patients with prostate cancer
Source: Clin Transl Med. 2023 Sep 12;13(9):e1411. doi: 10.1002/ctm2.1411 (PMC10497832; doi:10.1002/ctm2.1411)
Supplement: Supplementary file 1 — Supporting Information [file CTM2-13-e1411-s002.docx]

**Supplementary Methods**

**Study population**

In this multi-center cohort study, we consecutively enrolled unselected patients with PCa from Fudan University Shanghai Cancer Center (N = 1,910) and three other hospitals, including the Chinese University of Hong Kong Prince of Wales Hospital (N = 100), Sichuan University West China Hospital (N = 157), and Sun Yat-sen University Cancer Center (N = 148). All participants had a diagnosis of prostate adenocarcinoma. Clinical data was collected from medical records and test requisition forms, if available. 1,160 patients in the cohort have been reported previously^1^. Out of the initial cohort, 120 patients whose sequencing panels did not contain the genes of interest in this study and 143 patients with a missing personal or family history of cancer or metastatic status were excluded, leaving 2,127 patients as the development cohort (Figure S1). For the validation of our findings, we utilized two independent cohorts consisting of 920 PCa patients from Singapore and 549 PCa patients from Japan^2^, respectively. However, due to the lack of available cancer family history or metastatic status data, the final validation cohort was limited to 743 patients (396 from Japan and 347 from Singapore) (Figure S1). The study was approved by the ethics committees at each participating hospital, and written informed consent was obtained from each participant.

**Phenotype data**

The selection of potential risk factors for the development of APGRC was based on a comprehensive approach that considered published literature, clinical expertise, and pathophysiological reasoning. In this study, we collected phenotypic data, including age at diagnosis, family cancer history, personal cancer history, prostate specific antigen (PSA) level at diagnosis, Gleason score, clinical stage, and risk group. The risk group was determined based on the initial risk stratification for clinically localized disease as outlined by the National Comprehensive Cancer Network (NCCN)^3^. The data was collected under the conditions of regular clinical care, and its retrospective use for scientific purposes was approved by the ethics committee.

**Sample processing, DNA extraction, and quantification**

Peripheral blood samples were obtained from patients at all stages of their treatment and processed within a 72-hour window post-collection using Streck Cell-Free DNA BCT® tubes maintained at ambient temperature. The separation of plasma and buffy coat was accomplished through centrifugation of whole blood at 1600g for 10 minutes, followed by a secondary centrifugation of the resulting supernatant at 16000g for an additional 10 minutes. Buffy coat and liquid biopsy specimens were then preserved at -80℃ until the initiation of DNA extraction.

Germline DNA (gDNA) extraction was performed utilizing the DNeasy Blood & Tissue Kit (Qiagen, Hilden, Germany), adhering strictly to the manufacturer's stipulated protocol. DNA concentration was determined via the application of a Qubit fluorometer 3.0 coupled with the Qubit dsDNA High Sensitivity (HS) Assay Kit (Invitrogen, Carlsbad, CA, USA). The size distribution of DNA fragments was assessed employing an Agilent 2100 Bioanalyzer in conjunction with the DNA HS Kit (Agilent Technologies, Santa Clara, CA, USA). The criteria for sample quality necessitated gDNA with fragments exceeding 1000 base pairs in length.

**Library preparation, sequencing, quality assurance, and quality control**

A total of 100 ng of gDNA was subjected to shearing via the Covaris E210 system (Covaris), yielding fragments of approximately 200 base pairs in size. All DNA specimens underwent library preparation utilizing the Accel-NGS 2S DNA Library Kit (Swift Biosciences) combined with the xGen Lockdown Probes kit (IDT). The multigene panel used ranged from 2 to 1,460 genes, or alternatively, whole-exome sequencing was performed at the discretion of the ordering clinician. The customized xGen Lockdown probes, synthesized by IDT, Inc., were specifically tailored to target exonic and selected intronic regions of panel genes.

The custom-designed xGen Lockdown probes were adept at detecting mutations, as well as small insertions and deletions. Post-library preparation, precise quantification was achieved through the application of the Qubit 3.0 Fluorometer. Additional assessments encompassed the evaluation of quality attributes and fragment sizes, a task accomplished via the Agilent 2100 Bioanalyzer (reference fragment size: 280-350 bp; DNA quality: 0.5-50 ng/ul). Samples underwent paired-end sequencing on an Illumina Novaseq 6000 platform (Illumina) with 2 x 150-bp read length. The average sequencing depth was over 200× coverage for all participants in the development cohort and 100× coverage in the validation cohort.

**Data processing and Quality control**

The initial raw sequencing data underwent alignment to the GRCh37/hg19 reference genome utilizing the Burrows-Wheeler Aligner. Subsequent to the elimination of duplicate reads and local realignment procedures, the Genome Analysis Toolkit and LoFreq were utilized for the purpose of single nucleotide variation, and short insertions/deletions calling. The resulting variants were subsequently subjected to comprehensive annotation through utilization of the ANNOVAR software tool.

**Germline variants analysis and annotation**

In this study, a 14-gene panel (comprising of *ATM*, *BRCA1*, *BRCA2*, *BRIP1*, *CHEK2*, *FANCA*, *NBN*, *PALB2*, *RAD51C*, *RAD51D*, *MLH1*, *MSH2*, *MSH6*, and *PMS2*) was constructed after a comprehensive literature review^3-9^, which encompasses the genes recommended by major guidelines for testing in prostate cancer patients. Candidate variants identified in gDNA were determined as the valid germline variants for further analysis if they met the following criteria: (1) the allele frequency (AF) was beyond 30%; (2) supporting reads of the allele and variant were at least 15 and 8, respectively; (3) the frequency of the variants was below 1% in the public single-nucleotide polymorphism databases, including 1000 genomes (https://www.1000genomes.org/), ESP6500 (https://evs.gs. washington.edu/), ExAC (http://exac.broadinstitute.org/) and gnomAD (https://gnomad.broadinstitute.org/); (4) the variants were not synonymous SNV; (5) the variants were in the exon or splicing site; (6) the variants were not present in the inhouse repeat sequence database based on > 10000 cancer patients and healthy men. The clinical significance of the identified germline variants was evaluated based on the standards of the American College of Medical Genetics and Genomics and the Association for Molecular Pathology (ACMG/AMP)^10^, with the aid of InterVar^11^. In the absence of expert panel results, the consensus classifications in ClinVar were referred to. This study considered only pathogenic/likely pathogenic variants to be deleterious for further analysis.

Given that our patient samples originate from multiple centers, our sequencing was conducted across these various centers. To ensure the consistency and reliability of sequencing outcomes, we diligently followed a standardized operational protocol. Employing identical reagents, equipment, and consistent quality control criteria across all centers bolstered our efforts. This rigorous approach guarantees the uniformity of our sequencing results, aligning with the requirements of a multi-center study and fostering the validity of our findings.

**Model development**

To identify which clinical parameters could predict the presence of germline PVs in 14 PCa predisposition DDR genes, univariate logistic regression analyses were performed. All variables that were found to have a significant association with mutation status (p < 0.05) were considered for inclusion in the stepwise multivariate logistic regression analysis. The best model was selected based on the Akaike information criteria (AIC). To avoid the presence of collinear variables, the variables with significant collinearity were not included in the stepwise multivariate analysis at the same time. A nomogram was then created using the results of the stepwise multivariate logistic regression analysis and by using the rms package in R, version 4.2.2 (http://www.r-project.org/). We developed a website interface (https://apgrc.shinyapps.io/APGRC/), which allows for ease of access and potential future extensions of the APGRC model (Figure S4).

**Evaluation of the predictive performance of the model**

The performance of the nomogram was evaluated using multiple metrics. The discrimination of the nomogram was measured by the area under the receiver operating characteristic curve (AUC) in both the development cohort and validation cohort^12^. The calibration of the model was assessed using the Hosmer-Lemeshow test, and a p-value greater than 0.05 indicated good calibration. To evaluate the clinical utility of the nomogram, decision curve analysis (DCA) was conducted to estimate the net benefit of the model, which was defined as the proportion of true positives minus the proportion of false positives, considering the relative harm of false-negative and false-positive results^13,14^. The nomogram was further validated using 1,000 bootstrap resamples for internal validation, to obtain more accurate estimates of its performance.

**Missing data imputation**

To enhance the robustness and accuracy of the model, we excluded patients lacking key clinical information, including metastasis status, personal cancer history, and family cancer history (Figure S1). However, we tolerated the missing data of age, PSA, Gleason score, T stage, and nodal involvement by handling the missing data through imputation, thus preventing data loss. The imputation of missing data was performed using the random forest algorithm's proximity, which accounts for both numerical continuous variables and categorical variables. For continuous predictors, the imputed value was the weighted average of the non-missing observations, with the weights being the proximities. For categorical predictors, the imputed value was the category with the highest average proximity.

**Statistical analysis**

In this study, continuous data were presented as medians and interquartile ranges (IQR), while categorical data were represented as frequencies and percentages. The statistical analyses were conducted using R software (version 4.2.0), and a significance level of P < 0.05 was used to determine statistical significance.

1. Zhu Y, Wei Y, Zeng H, et al. Inherited Mutations in Chinese Men With Prostate Cancer. *J Natl Compr Canc Netw.* 2021;20(1):54-62.

2. Kimura H, Mizuno K, Shiota M, et al. Prognostic significance of pathogenic variants in BRCA1, BRCA2, ATM and PALB2 genes in men undergoing hormonal therapy for advanced prostate cancer. *Br J Cancer.* 2022;127(9):1680-1690.

3. Carlo MI, Giri VN, Paller CJ, et al. Evolving Intersection Between Inherited Cancer Genetics and Therapeutic Clinical Trials in Prostate Cancer: A White Paper From the Germline Genetics Working Group of the Prostate Cancer Clinical Trials Consortium. *JCO Precis Oncol.* 2018;2018.

4. Lowrance WT, Breau RH, Chou R, et al. Advanced Prostate Cancer: AUA/ASTRO/SUO Guideline PART I. *J Urol.* 2021;205(1):14-21.

5. Lowrance WT, Breau RH, Chou R, et al. Advanced Prostate Cancer: AUA/ASTRO/SUO Guideline PART II. *J Urol.* 2021;205(1):22-29.

6. Eastham JA, Boorjian SA, Kirkby E. Clinically Localized Prostate Cancer: AUA/ASTRO Guideline. *J Urol.* 2022;208(3):505-507.

7. Mottet N, Cornford P, Bergh RCNvd, et al. EAU-EANM-ESTRO-ESUR-ISUP-SIOG guidelines on prostate cancer. 2022.

8. Cheng HH, Sokolova AO, Schaeffer EM, Small EJ, Higano CS. Germline and Somatic Mutations in Prostate Cancer for the Clinician. *J Natl Compr Canc Netw.* 2019;17(5):515-521.

9. Giri VN, Knudsen KE, Kelly WK, et al. Role of Genetic Testing for Inherited Prostate Cancer Risk: Philadelphia Prostate Cancer Consensus Conference 2017. *J Clin Oncol.* 2018;36(4):414-424.

10. Richards S, Aziz N, Bale S, et al. Standards and guidelines for the interpretation of sequence variants: a joint consensus recommendation of the American College of Medical Genetics and Genomics and the Association for Molecular Pathology. *Genet Med.* 2015;17(5):405-424.

11. Li Q, Wang K. InterVar: Clinical Interpretation of Genetic Variants by the 2015 ACMG-AMP Guidelines. *Am J Hum Genet.* 2017;100(2):267-280.

12. Hanley JA, McNeil BJ. The meaning and use of the area under a receiver operating characteristic (ROC) curve. *Radiology.* 1982;143(1):29-36.

13. Balachandran VP, Gonen M, Smith JJ, DeMatteo RP. Nomograms in oncology: more than meets the eye. *Lancet Oncol.* 2015;16(4):e173-180.

14. Collins GS, Reitsma JB, Altman DG, Moons KG. Transparent Reporting of a multivariable prediction model for Individual Prognosis Or Diagnosis (TRIPOD). *Ann Intern Med.* 2015;162(10):735-736.
