# Supplementary material for: Development and validation of a nomogram (APGRC) to predict the presence of germline DNA damage repair pathogenic variants in Asian patients with prostate cancer
Source: Clin Transl Med. 2023 Sep 12;13(9):e1411. doi: 10.1002/ctm2.1411 (PMC10497832; doi:10.1002/ctm2.1411)
Supplement: Supplementary file 2 — Supporting Information [file CTM2-13-e1411-s003.docx]

**Supplementary figure 1: Flowchart**


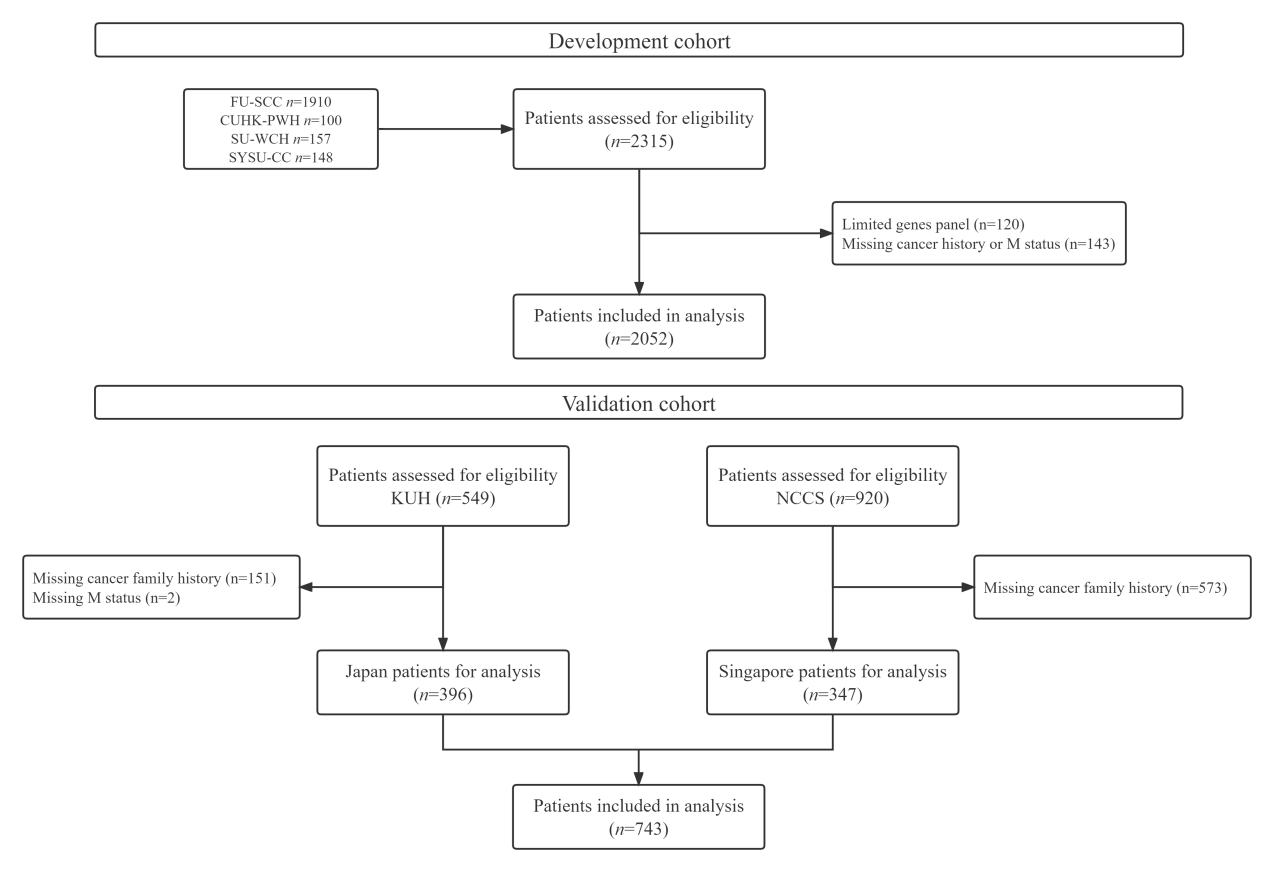


Abbreviations: FU-SCC, Fudan University Shanghai Cancer Center; CUHK-PWH, the Chinese University of Hong Kong Prince of Wales Hospital; SU-WCH, the Sichuan University West China Hospital; SYSU-CC, the Sun Yat-sen University Cancer Center; M, metastasis; KUH, Kyoto University Hospital; NCCS, National Cancer Centre Singapore

**Supplementary figure 2: Pathogenic variant frequencies in 14 PCa predisposition DDR genes among cohorts**


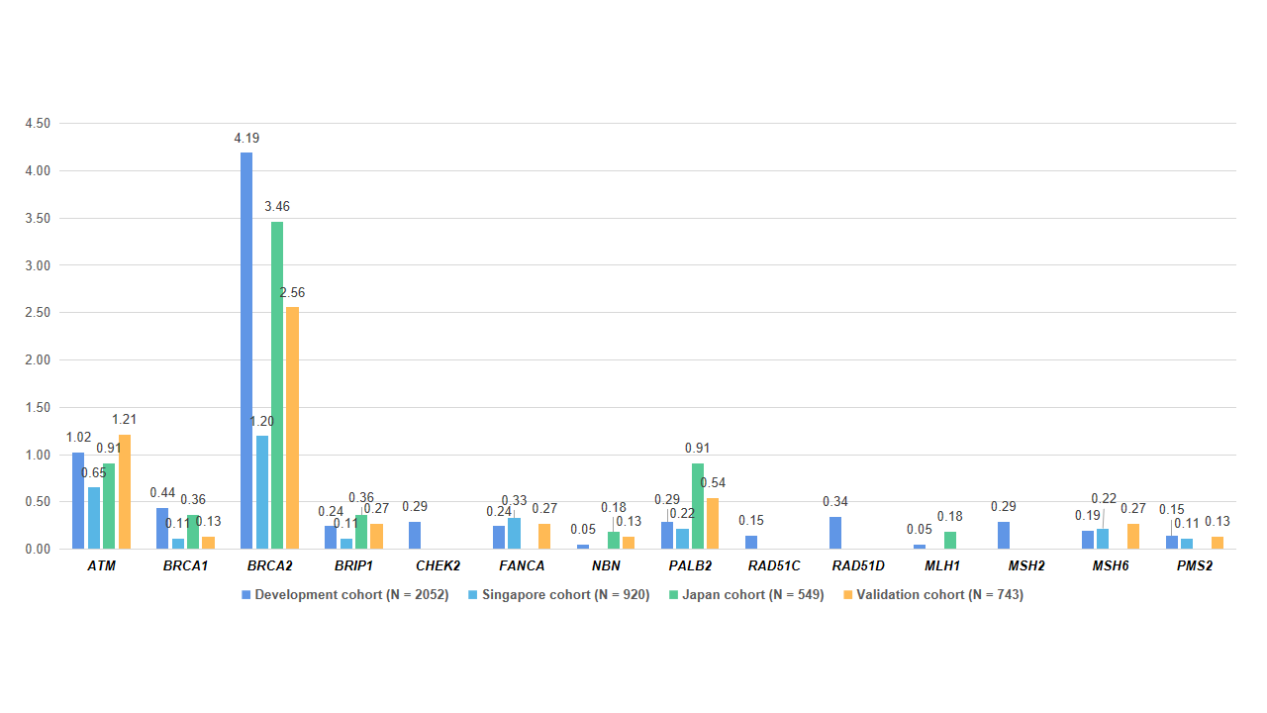


**Supplementary figure 3: The contribution of age at diagnosis to the APGRC model**


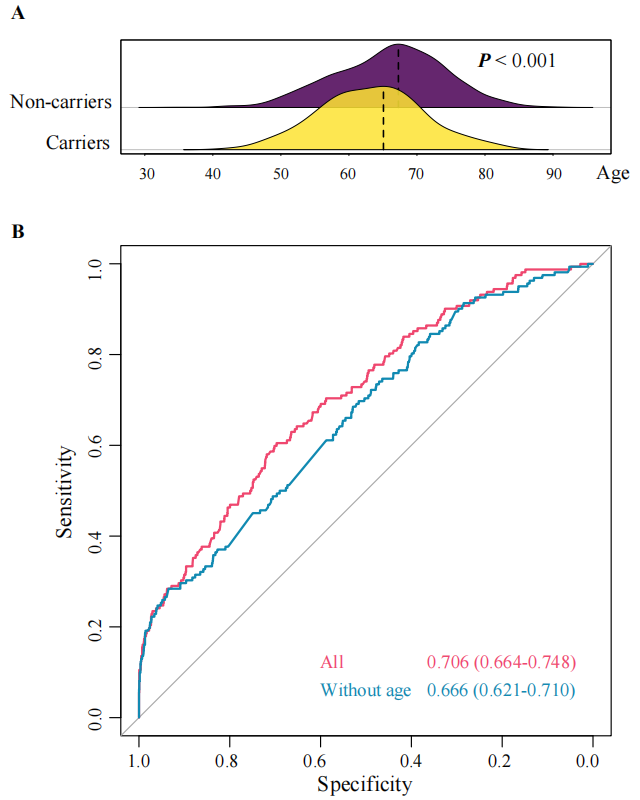


**Supplementary figure 4: The interface of the online APGRC tool**


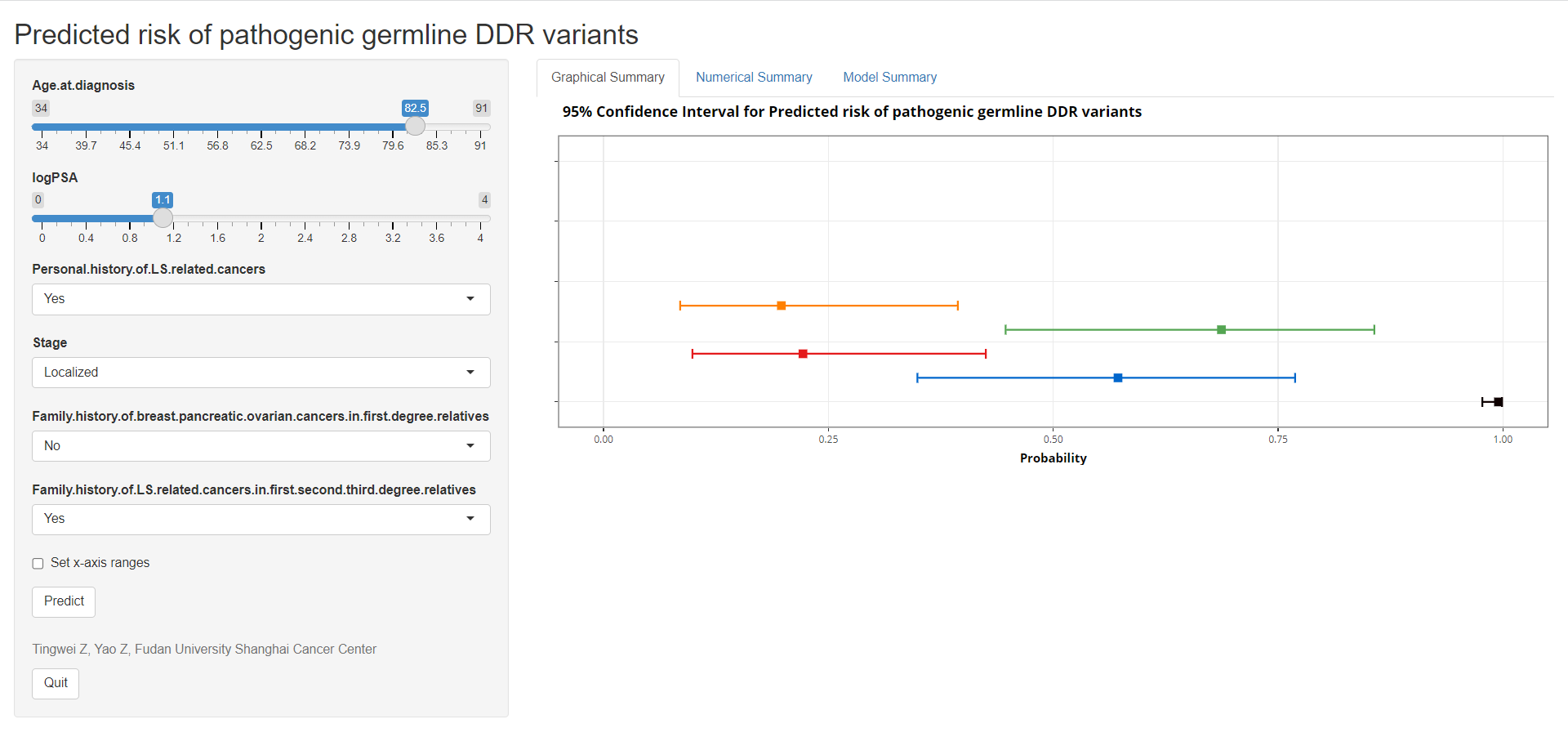


For the online APGRC tool: https://apgrc.shinyapps.io/APGRC/

Abbreviations: PSA, prostate specific antigen; DDR, DNA damage repair; LS, Lynch Syndrome
